# Multistability manipulation by reinforcement learning algorithm inside mode-locked fiber laser

**DOI:** 10.1515/nanoph-2023-0792

**Published:** 2024-04-15

**Authors:** Alexey Kokhanovskiy, Evgeny Kuprikov, Kirill Serebrennikov, Aram Mkrtchyan, Ayvaz Davletkhanov, Alexey Bunkov, Dmitry Krasnikov, Mikhail Shashkov, Albert Nasibulin, Yuriy Gladush

**Affiliations:** School of Physics and Engineering, 65071 ITMO University, St. Petersburg 197101, Russia; 64941 Novosibirsk State University, Pirogova 2, Novosibirsk 630090, Russia; 366033 Skolkovo Institute of Science and Technology, Moscow 121205, Russia; 104675 Boreskov Institute of Catalysis SB RAS, Novosibirsk 630090, Russia; Institute of Automation and Electrometry SB RAS, 1 Ac. Koptyug ave., Novosibirsk 630090, Russia

**Keywords:** multistability, harmonic mode-locked lasers, reinforcement learning, single wall carbon nanotubes, saturable absorber

## Abstract

Fiber mode-locked lasers are nonlinear optical systems that provide ultrashort pulses at high repetition rates. However, adjusting the cavity parameters is often a challenging task due to the intrinsic multistability of a laser system. Depending on the adjustment of the cavity parameters, the optical output may vary significantly, including Q-switching, single and multipulse, and harmonic mode-locked regimes. In this study, we demonstrate an experimental implementation of the Soft Actor–Critic algorithm for generating a harmonic mode-locked regime inside a state-of-the-art fiber laser with an ion-gated nanotube saturable absorber. The algorithm employs nontrivial strategies to achieve a guaranteed harmonic mode-locked regime with the highest order by effectively managing the pumping power of a laser system and the nonlinear transmission of a nanotube absorber. Our results demonstrate a robust and feasible machine-learning–based approach toward an automatic system for adjusting nonlinear optical systems with the presence of multistability phenomena.

## 1 Introduction

The term multistability refers to the property of a physical system to possess multiple stable states for a given set of system parameters [1]–[3]. Such behavior can be demonstrated by a wide variety of complex dissipative systems, including lasers [4]–[8], spin ensembles [9]–[11] and many others [12], [13]. For the majority of applications, the phenomenon of multistability is a detrimental outcome that diminishes the system’s robustness and predictability. However, smart exploitation of multistability reveals new approaches to controlling the systems [14], [15] and opens new ways of creating novel devices.

Mode-locked fiber lasers include key components for multistability: nonlinearity, energy flow and dissipation, feed-back loop, and fluctuations [16]. The dynamic of this system is governed by generalized Ginzburg–Landau equation, incorporating self-phase modulation, saturable absorption, and gain saturation as well as dispersion complemented by higher order nonlinear and dispersion terms in specific cases. The pulse shape is usually associated with the famous soliton solution, referred as conservative soliton in laser physics [17]; strictly speaking, it is valid only for pulse propagation in uniform single mode fiber. In real laser cavity, the pulse is subject to permanent perturbations as it travels from one optical element to another giving rise to such attractors, as dissipative solitons [18] and dispersion managed solitons [19]. The final pulse parameters are a result of dynamic equilibrium and will be dependent not only on average laser parameters, such as pump power, dispersion, and nonlinearity, but also on the elements order in the laser [20] or previous state of the laser generation, manifesting in hysteresis behavior. The later one appears for the threshold power on transition between continuous wave (CW) and mode-locked generation [21], [22]. Further increase of the intracavity energy can lead to existence of new types of solutions including multipulse generation, harmonic mode locking (HML), soliton rains [23], and rogue waves [24]. Among them, HML, corresponding to formation of equidistant sequence of pulses in cavity, holds great significance for the applied science due to the ability to generate ultrashort pulse trains with up to hundreds of gigahertz repetition rates [25]–[27].

There are several approaches for qualitative explanation for HML regimes, including interaction between separated solitons through a dispersive wave leading to binding or repulsion of the solitons [28], [29] and interaction through slow gain saturation [30]–[33]. In practice, these interactions are weak and cannot be controlled directly. For decades, researchers have demonstrated experimental platforms for HML generation, including lasers with saturable absorbers [34], fiber loop mirrors [35], photonic crystal fibers [36], and Mach–Zehnder interferometer [37]. The majority of the sources were based on manipulation with the states of polarization controllers, representing a blind search method [28], [38]–[40]. It was shown that by manipulating the state of polarization controller inside a fiber cavity, different orders of HML regimes may be achieved at the same pump power. Even more, recovering the same polarization controller position does not necessary lead to the same HML regime. This ambiguity, together with the environmental sensitivity of nonpolarization-maintaining fibers, makes this approach inappropriate for commercial use.

To address this ambiguity and stability issues, various machine-learning approaches, commonly referred to as universal approximators, were introduced, which have demonstrated efficiency in finding and stabilizing regime of interest [41]. The majority of experimental realizations address the intracavity polarization state manipulation through electronic polarization controllers based on genetic or evolutionary algorithms [42]–[45]. Today, the special focus of the community is under the special paradigm of machine-learning algorithms – reinforcement learning (RL) [46]. These algorithms are capable of making sequential decisions to solve the task after a training procedure. For mode-locked lasers, there are already promising applications for RL-algorithms to control the output radiation including stabilizing mode-locked regime from environmental disturbance [47], [48], adjusting numerical model of mode-locked laser though bi-stability [49]. RL-algorithms are more general and robust than popular evolutional algorithms, which are widely employed for adjusting mode-locked lasers [48]. They do not search for a maximum value at static space of a fitness function, but instead find an optimized strategy for dynamic adjustments of controlling elements. While the manipulation through polarization state has been widely explored, investigations of the applicability of machine-learning algorithms for more robust PM lasers are very rare.

Here, we propose an RL algorithm for searching the desired HML regime in a polarization-maintaining fiber laser. The algorithm treats the fiber laser as a black box, considering only the possibility of the laser operating at the HML regime and controlling a discrete number of parameters of the laser cavity. We applied a Soft Actor–Critic (SAC) algorithm capable of finding a dynamic strategy for controlling pump power and saturable absorption modulation depth to obtain HML generation of the maximum order. We deliberately chose a laser system with only two controlling parameters. Even for this simple system, the laser demonstrates multistable operation, converging to Q-switching or different orders of HML depending on its initial conditions, making manual search very time-consuming. The adjustment of our laser setup is not a simple 2D-optimization problem. The multistability phenomenon presents a considerable degree of complexity, as the laser’s output regime is dependent on its previous state in time, in addition to a set of controlling parameters. To achieve the highest order HML regime, one must optimize the trajectory in the parameter space with an arbitrary number of steps. The number and magnitude of the steps are not known in advance and they are dependent on the current state of the laser. Such an optimization task is difficult to formalize for an evolutionary algorithm and hardly achievable with a simple grid-search technique.

## 2 Experimental setup

**Figure 1:**
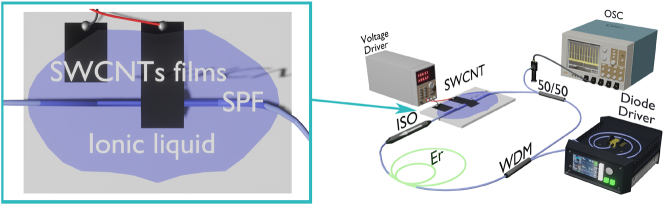
Experimental setup of a mode-locked fiber laser and measuring system.

We implemented an all-polarization-maintaining (all-PM) fiber laser with a ring cavity design, which is schematically shown in Figure 1.

The all-PM scheme provided environmental stability to external perturbations and ensured that effects associated with nonlinear polarization evolution did not influence pulse generation. We utilized 0.6 m of highly doped large mode area erbium-doped fiber (EDF) LIEKKI Er80-8/125-PM as a gain medium with 80 dB/m absorption and anomalous dispersion 20.6 ⋅ 10^3^, fs^2^/m at the lasing wavelength. The EDF was excited by a 976 nm laser diode (LD) through a wavelength-division multiplexer composed of a fast axis blocked isolator (ISO) to ensure polarized unidirectional lasing. For the output, we used a 50:50 optical coupler. The total length and net dispersion of the fiber resonator were about 5.7 m and −0.13 ps^2^, respectively. A single-walled carbon nanotubes (SWCNT) film was utilized as a saturable absorber on a side-polished fiber (SPF). To manipulate the nonlinear absorption of the SWCNT film, we used electrochemical gating.

**Figure 2:**
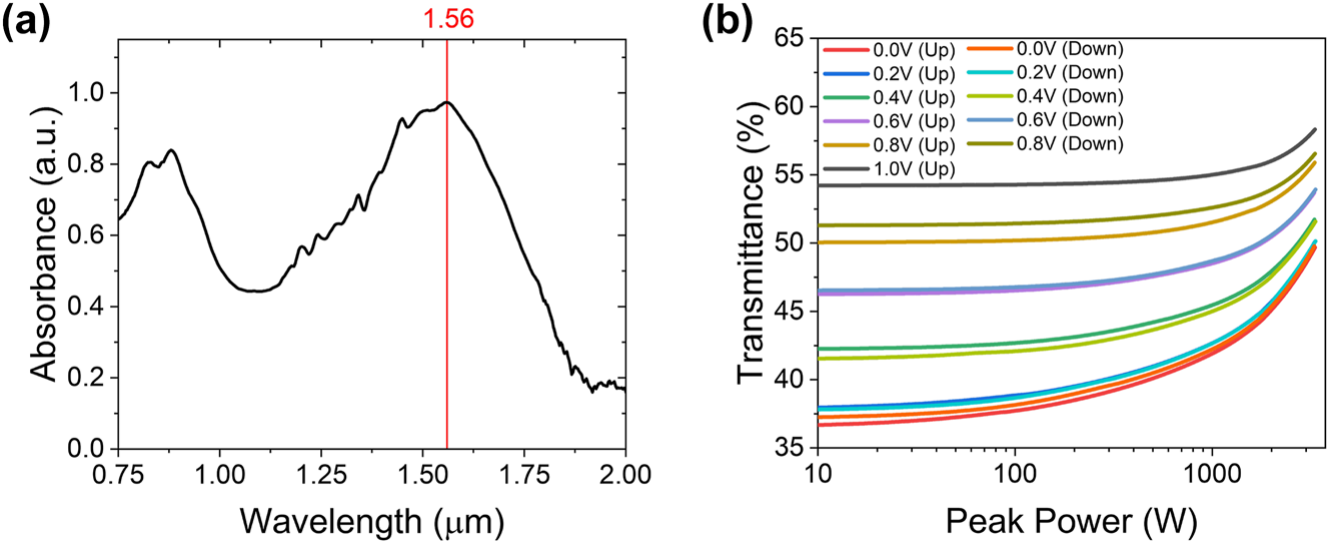
Optical properties of the SWNCNT film. (a) Relative absorption spectrum of the SWCNT film and (b) nonlinear transmission of the SWCNT film immerged to ion liquid for different values of applied voltage when voltage is increased from 0 V to 1 V (up) and decreased from 1 V to 0 V (down).

Single-walled carbon nanotubes were synthesized using the aerosol (floating catalyst) chemical vapor deposition method [50]. The mean diameter of the SWCNTs was directly adjusted during synthesis [51] to ensure resonance between the semiconducting *S*_11_ interband transition and the laser operating wavelength of 1.5 µm (see Figure 2a).

The carbon nanotube film was transferred to the polished surface of the SPF using the dry transfer technique [52]. The light polarization was chosen in the plane with the polished surface, ensuring strong interaction with SWCNTs. We then created an ionic liquid cell for electrochemical gating of SWCNTs, similar to our previous work [53] (see Figure 1 zoom-in). For this, we added a second carbon nanotube film as a counter electrode next to the SWCNTs film on SPF. Stable ionic liquid Bmim NTf_2_, operational under ambient conditions, was dripped to cover both SWCNT films [54]. By applying voltage to the film, we can shift the Fermi level of SWCNTs and, consequently, decrease the saturable absorber modulation depth. From Figure 2b, slight differences in small signal losses is observed for the measurement when increasing the voltage and decreasing it. This sensitivity to previous state can be explained by a difference in the potential required for the ions to enter the electrical double layer region and to leave it. To minimize this hysteresis behavior and to ensure the reproducible operation of the electrochemical cell, the voltage was always kept below 1 V. In previous work [53], we used SWCNT electrochemical gating to demonstrate switching between fundamental mode locking and Q-switching. Here, we applied it for higher pump powers where switching between different orders of HML is possible.

**Figure 3:**
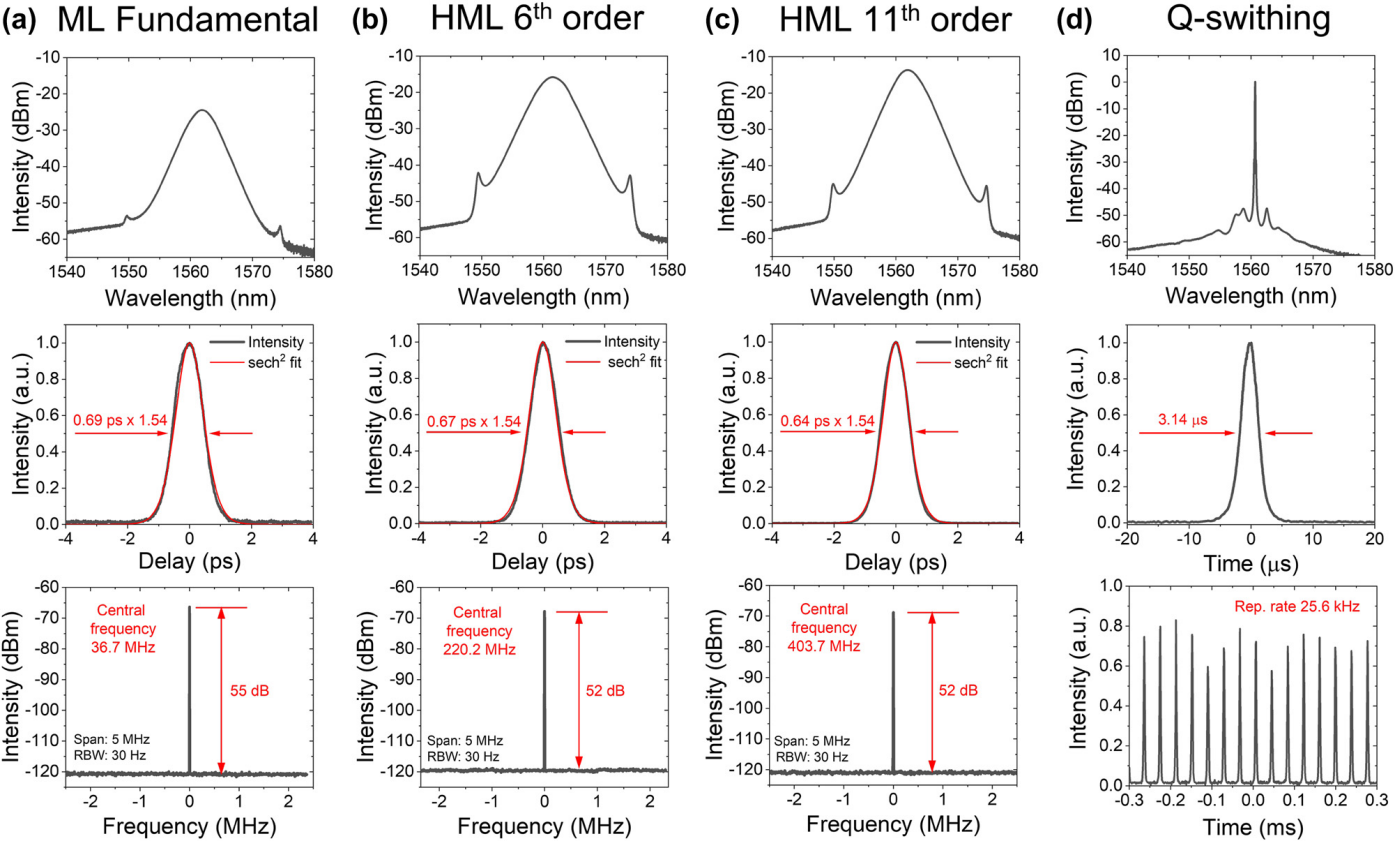
Optical spectrums, autocorrelation functions, and radio-frequency spectrums of the possible pulsed regimes generated inside the laser cavity, (a) mode-locked regime, (b) 6th-order harmonic mode-locked regime, (c) 11th-order harmonic mode-locked regime, and d) Q-switched regime.

The continuous-wave generation starts at 25 mW of the pump diode power. By increasing the pump power, self-starting fundamental pulse generation appeared at a 36.7 MHz repetition rate with a 690 fs pulse duration. The application of voltage on the electrochemical cell led to a Q-switch regime with a repetition rate in the tens of kHz and a µs-scale pulse duration. Further increase of the pump power led to multipulse generation or harmonic mode-locking with orders up to 11, with Q-switching separating every harmonic order (Figure 3). The radio-frequency peak of a single pulse mode-locked regime was measured in the vicinity of the fundamental mode of the laser cavity, and it was found to have a height of 55 dB, which is indicative of high-quality mode-locked regimes that do not require any special stabilization techniques. The radio-frequency peak of the 11th order HML regime decreased down to 52 dB, but still may be considered as low noise regime for large amounts of applications. We did not take special measurements of long-term stability of HML regimes; however, HML regimes adjusted by SAC algorithm were stable for at least several hours and days.

**Figure 4:**
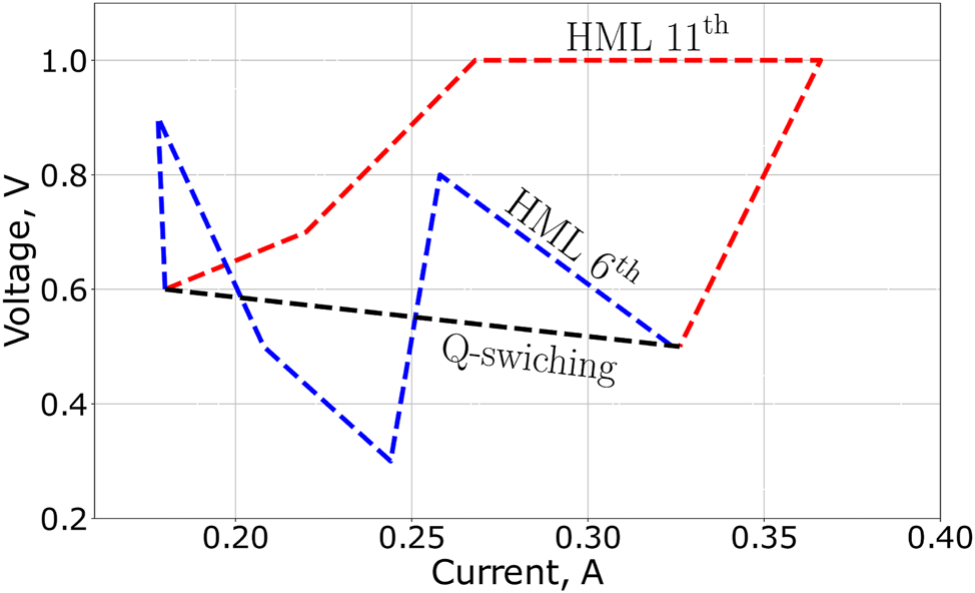
Dependence of the final type of the pulsed regime generated inside the laser cavity against trajectories of the adjusting pumping current of the laser diode and applied voltage on SWCNT film. Starting from the initial point (0.16 A, 0.6 V), one can achieve different pulsed regimes at (0.325 A, 0.5 V), including Q-switched regime, HML 6th and 11th order.

Application of voltage on electrochemical cell in these regimes could lead to increase of the number of pulses or to formation of harmonic mode locking out of multipulse generation, giving additional degree of freedom. Interestingly, the order of HML at particular pump power and voltage on the cell was dependent on the trajectory to this point in parameter space. An example is shown at Figure 4. A direct jump from fundamental ML regime to the final point led to Q-switching. In contrast, gradual increase of the pumping power along with small voltage variations led to 6th order HML generation. Increasing the voltage to 1 V along with the pumping power increase and then decreasing the voltage down to 0.5 V (red color in Figure 4) led to 11th order of HML at the same final voltage and pump parameters. In order to obtain the highest order HML regime, a certain speed of adjusting the cavity parameters must be maintained. Otherwise, Q-switch or unstable regimes may occur. This kind of behavior makes the problem of finding the maximum available order of HML a challenging task due to the large number of possible trajectories even in two dimensional parameter space. It required a period of time ranging from several minutes to an hour to manually adjust the HML regime with the highest order.

## 3 Reinforcement learning

### 3.1 Mode-locked fiber laser as RL environment

The RL algorithm operates with the fiber laser as a black-box. *A priori*, the algorithm lacks information about the underlying physics and learns the behavior of the system through actions and their consequences. In terms of the RL algorithm, we formulate the considered problem as follows: the laser serves as an environment where an agent – a neural network – acts by changing the parameters of the cavity. The fixed parameters of the laser cavity determine the characteristics of the optical output, denoted as the state of the environment. The goal for the agent is to maximize the reward gained for appropriate actions. The reward is a scalar function designed to have a maximum at the desired mode-locked regime. Changes to the reward function necessitate repeating the training procedure. The agent will be able to select actions based only on the current state of the system and can reach the goal from any starting point presented during the training. To calculate the reward function, we used the order of the HML regime $N_{\text{HML}}=\frac{F}{F_{f}}$ and the number of randomly spaced mode-locked pulses in the measured time scale *N*.

(1) $$R=N\frac{F}{F_{f}}$$

where *F*_*f*_ represents the fundamental frequency, and *F* is the repetition rate of the mode-locked regime. We used the product of the order of the HML regime and the number of pulses to assign a higher reward to multipulsing regimes, considering them as precursor regimes before the HML regim.

To describe the state *s* of the laser system, we utilized five values: the voltage applied to ionic liquid-gated SWCNTs, the current applied to the pump diode, the average and maximum values of the oscillogram trace, the repetition rate of a pulse train, and the number of pulses within the span of the oscilloscope trace. The action *a* is a vector of two values corresponding to the steps of adjusting voltage applied to the nanotubes and current applied to the pumping diode. The agent receives the state *s* of the system as input and outputs a vector of two values in the continuous interval [−1, 1], with each value scaled by *V*_*V*_ = 1 and *V*_*I*_ = 0.1 for voltage and current, respectively. The resulting values are then added to the real values of the controlling devices. The applied voltage on the SWCNTs cell was varied in the range from 0 to 1 V and the current in the range from 100 to 400 mA.

The transition time from one state of the laser to another is determined by the stabilization time of a pulsed regime after the cavity parameters were changed. We have used radio-frequency spectrum of the output radiation to verify the stability of the pulse regime. We applied Fourier transformation to an oscilloscope trace of the pulse train and measured the amplitude of the peak corresponding to the repetition rate of the pulse train. After adjusting the cavity parameters, the amplitude of the peak was measured once per second until the last two measurements showed a difference of less than 5 %. Stabilization of a pulsed regime took 12 s on average. The maximum waiting time was set to 20 s for nonstable regimes, such as multipulsed regimes with moving solitons against each other in time. After that, the last measurement of the oscilloscope trace was taken as the current state. If the pulsed regime was lost or a Q-switch state was generated, the attempt to adjust by the RL algorithm ended.

### 3.2 Soft Actor–Critic

**Figure 5:**
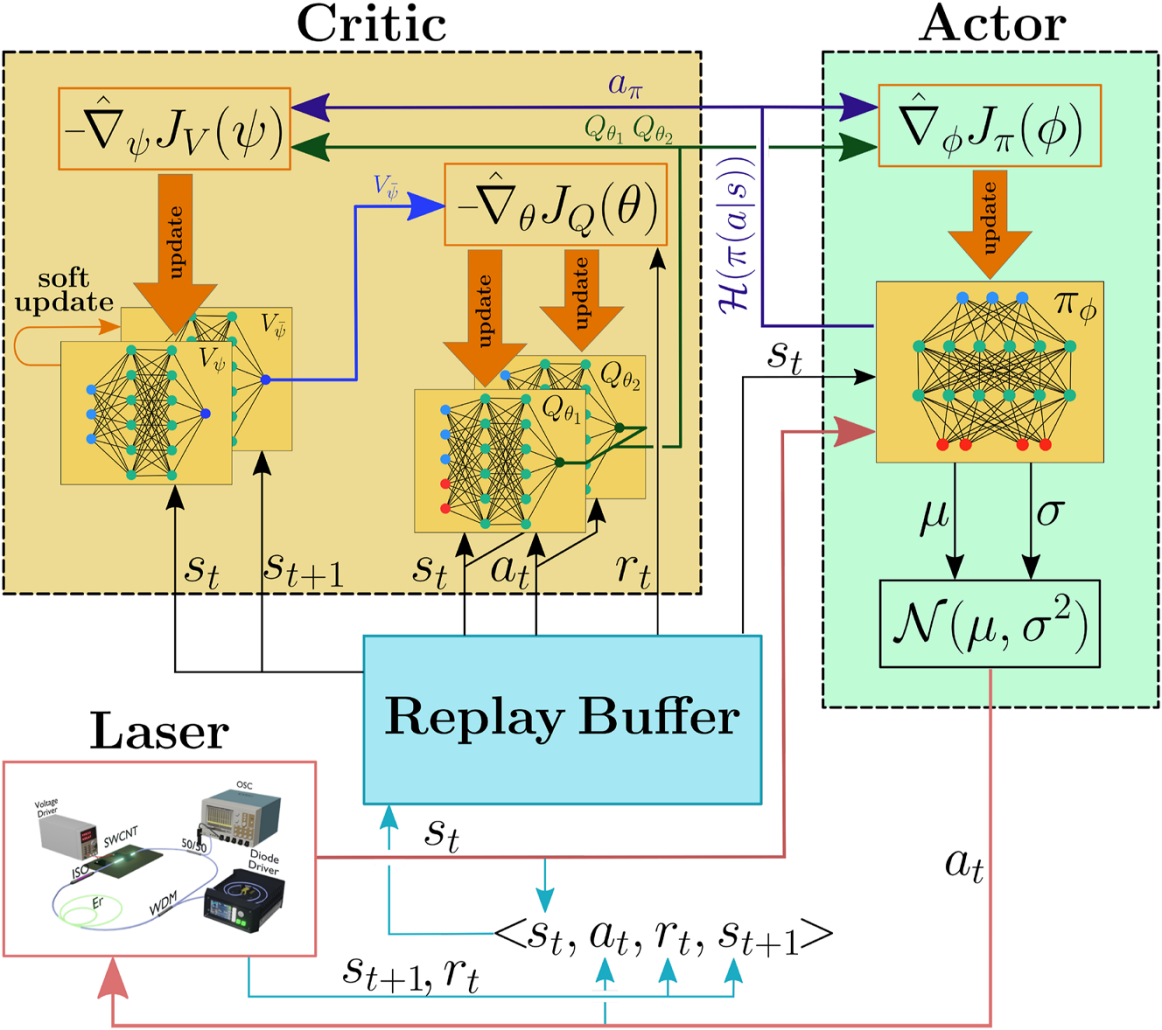
Schematic diagram of the Soft Actor–Critic algorithm. The agent consists of two parts: Actor and Critic. Actor is a feed-forward neural network and Critic is a set of four connected feed-forward neural networks. The Actor is trained to adjust laser cavity parameters through the sequence of actions (red arrow). The Critic is trained to evaluate the Actor’s policy (violet arrow) and retrain Actor (green arrow). The experience replay buffer, a record of transitions from one state to another through the action, is used to train the Actor on old experiences (black arrow). The light blue arrows show the process of collecting data into the buffer.

Soft Actor–Critic (SAC) was developed to train the agent on choosing the next step from a continuous space of actions. The algorithm separates the agent into two distinct components: the Actor and the Critic (Figure 5). The Actor determines which action to take based on a policy, denoted as *π*(*a*|*s*). In practice, *π*(*a*|*s*) represents the conditional probability of choosing action *a* in state *s*. The Critic evaluates the current policy of the Actor and prompts improvements. The key innovation of the SAC algorithm involves reward modification through the addition of entropy regularization.

(2) $$r_{\text{soft}}\left(s,a\right)=r\left(s,a\right)+\alpha H\left(\pi \left(a|s\right)\right),$$

where *r*(*s*, *a*) is the reward obtained by performing an action *a* in a state *s*, H(π(a|s)) is the entropy of the policy *π* at state *s*, and *α* is a hyperparameter known as temperature, determining the relative importance of the entropy term against the reward. To prevent the policy from becoming deterministic with a degenerate distribution, the entropy of the policy *π*(*a*|*s*) is added to the reward received from the environment. This reward modification “smooths” the policy; hence, the algorithm is named “soft” actor–critic.

The SAC algorithm uses a normal distribution to describe the policy $\pi \left(a|s\right)=N\left(\mu \left(s\right),\sigma {\left(s\right)}^{2}\right)$ , where *μ*(*s*) and *σ*(*s*) – is the mean and standard deviation which depend on *s*. The goal of the algorithm is to find optimal parameters of the policy for each possible state. Due to the high dimensionality of the state space, artificial neural networks (ANN) are used for approximation. The Actor consists of ANN *π*_*ϕ*_ with parameters *ϕ*, which predicts the values of *μ* and *σ* for the state *s*, and the action *a* is chosen according to $N\left(\mu ,\sigma^{2}\right)$ .

In RL algorithms, the evaluation of the Actor’s policy involves the use of value functions. The state-value function *V*(*s*) indicates the profitability of being in state *s*, and the action-value function *Q*(*s*, *a*) reveals how profitable it is to take action *a* in state *s*. Profitability is determined by the expected sum of rewards that the agent can receive if it continues to act according to the policy *π*. These two value functions are interrelated through the Bellman equations [55]. Soft Bellman equations, considering a soft reward as given by Equation (2), take the following form [56].

(3) $$Q_{\text{soft}}\left(s,a\right)=r\left(s,a\right)+\gamma E_{s^{\prime }}\left[V_{\text{soft}}\left(s^{\prime }\right)\right],$$

(4) $$V_{\text{soft}}\left(s\right)=E_{\pi \left(a|s\right)}\left[Q_{\text{soft}}\left(s,a\right)+\alpha H\left(\pi \left(a|s\right)\right)\right],$$

were *s*′ is the next state, *γ* ∈ [0, 1] is the discount factor, which determines the value of future rewards in terms of present rewards, $E_{s^{\prime }}$ means the mathematical expectation for the next state *s*′, and $E_{\pi \left(a|s\right)}$ – the mathematical expectation by action *a* according to the policy *π*(*a*|*s*). These equations are written under the assumption that the entropy term is given at the moment of transition to state *s*, and after choosing action *a* the reward *r*(*s*, *a*) is given.

ANNs *Q*_*θ*_ and *V*_*ψ*_ are used to approximate the soft value functions. The learning process is to find *θ* and *ψ* that *Q*_*θ*_(*s*, *a*) and *V*_*ψ*_(*s*) satisfy the soft Bellman equations (3) and (4) for any step from *s* to *s*′ that was obtained during the agent’s interaction with the environment. For this purpose, all steps are collected in a buffer called the experience replay buffer, as shown in Figure 5. The one-step evaluation of the value functions is biased, so two standard heuristics, named target network [57] and clipped double estimation [58], are used to stabilize learning. The target network $V_{\bar{\psi }}$ , which is a copy of the network *V*_*ψ*_, is used to train *Q*_*θ*_. To update the weights of the $V_{\bar{\psi }}$ network, we used the soft update rule, which smoothly updates $\bar{\psi }$ to *ψ* like $\bar{\psi }=\left(1-\tau \right)\bar{\psi }+\tau \psi$ , where *τ* ≪ 1. The Critic tends to overestimate the values of the Q-function, so a clipped double estimation is used. For this two networks with weights, *θ*_1_ and *θ*_2_ are trained independently and the minimum of two estimates is used for training. Figure 5 shows these heuristics in detail.

According to the soft Bellman equations, the loss functions *J* for the networks *Q*_*θ*_ and *V*_*θ*_ are follows:

(5) $$J_{Q}\left(\theta \right)={\left[r+\gamma V_{\bar{\psi }}\left(s^{\prime }\right)-Q_{\theta }\left(s,a\right)\right]}^{2},$$

(6) $$J_{V}\left(\psi \right)={\left[\underset{i=1,2}{\min}Q_{\theta_{i}}\left(s,a_{\pi }\right)+\alpha H\pi \left(a|s\right)-V_{\psi }\left(s\right)\right]}^{2},$$

where the values $<s,a,r,s^{\prime }>$ are taken from the replay buffer and *a*_*π*_ is generated by the current policy according to *π*(*a*|*s*). A stochastic gradient descent method is used to train the networks. Figure 5 visualizes the design of the Critic, which consists of four ANNs. The black arrows show how the values from the replay buffer are used during training.

Finally, the following function is maximized to improve the Actor’s policy via improving Critics evaluation:

(7) $$J_{\pi }\left(\phi \right)=E_{\pi \left(s|a\right)}\underset{i=1,2}{\min}Q_{\theta_{i}}\left(s,a\right)+\alpha H\left(\pi \left(a|s\right)\right)\to \max_{\pi }.$$

Since the policy *π*(*a*|*s*) is stochastic, a reparameterization trick [59] is needed to make the target function differentiable. Reparameterization allows using the stochastic gradient ascent method to train the policy network.

In this work, the scripts used for remote control and measurements were written in Python. We used Pytorch framework to implement the SAC algorithm [60]. Each neural network architecture consisted of two hidden layers of 256 neurons. Discount factor *γ* = 0.99. For each network except $V_{\bar{\psi }}$ , an Adam optimizer with a learning parameter of 3 × 10^−4^ was used. For one update of the ANN’s weights, we used a set of samples $<s,a,r,s^{\prime }>$ with size 256 from the replay buffer. The $V_{\bar{\psi }}$ was updated by soft update rule with the parameter 0.05. Equation (2) shows that *α* strongly depends on the reward value *r*, so we normalize the reward values leaving *α* = 1. In this paper, *r* = *R*/100. So *r* = 1 corresponds to the 10th order of HML.

## 4 Results and discussion

**Figure 6:**
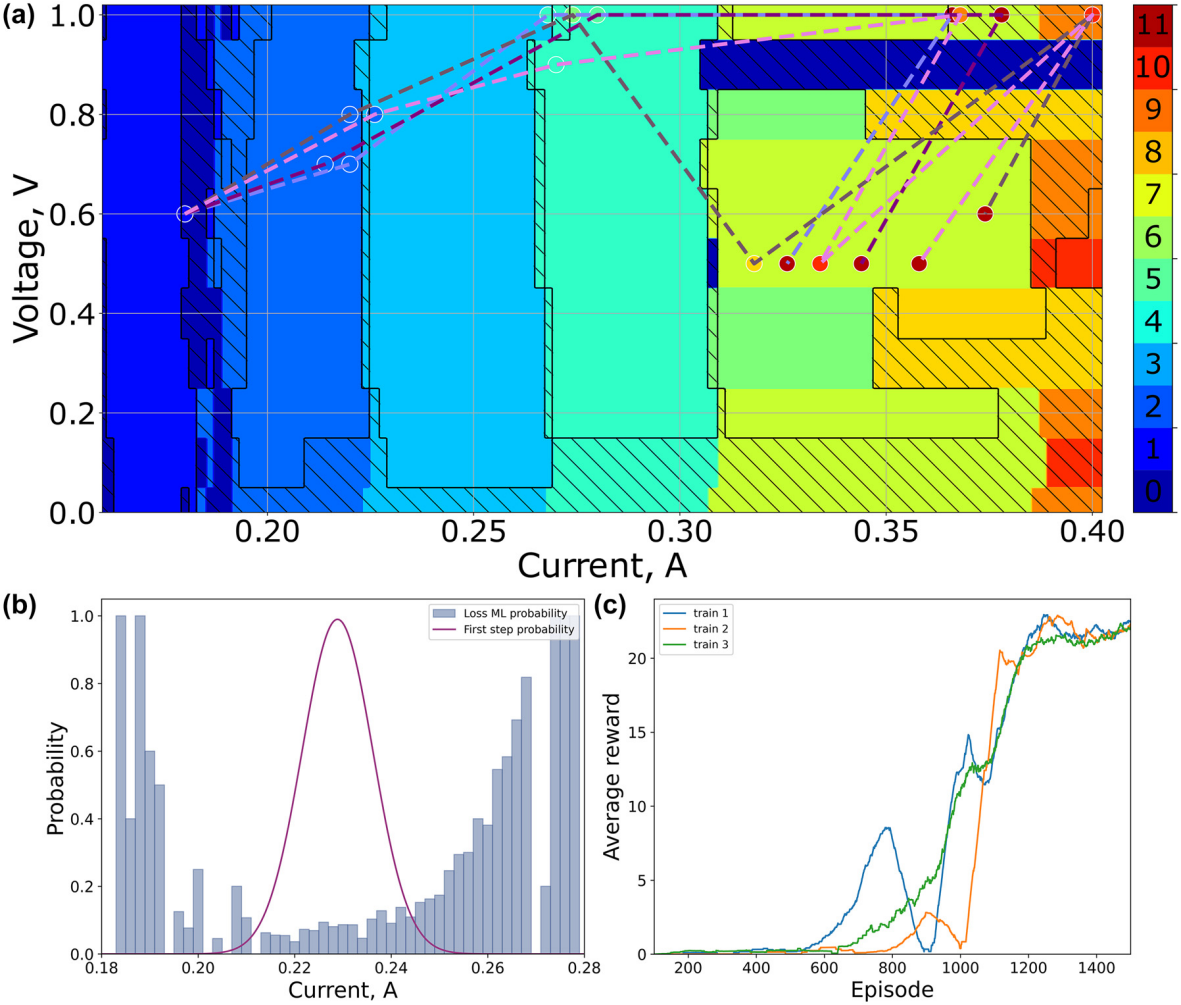
Performance of the SAC algorithm, (a) map of generation regimes, which was obtained by scanning the area. The color indicates the number of pulses in the regime. Solid filled areas indicate HML regimes. Oblique hatching corresponds to multipulsing regimes. The final trajectories of the agent leading to the maximum order of the harmonic mode-locked regimes is represented by the dashed lines. The color of the circles corresponds to the order of the harmonic mode-locked regime according to the colorbar. (b) Probability of making the first step by current. The histogram shows the probability of losing mode-locked regime. The purple line shows the distribution of the first step (scaled distribution). (c) The learning curves of the SAC algorithm for three independent train procedures.

Before commencing the training of the SAC algorithm, we generated a map of the laser’s output states, as depicted in Figure 6a. The map was acquired in the following sequence: at a fixed voltage on the SWCNTs film, starting from 0 V, we systematically increased the diode current from 160 to 400 mA with a step of 1 mA, measuring the oscilloscope trace at each step. Subsequently, the diode current was decreased back to 160 mA, while the voltage was incremented by 0.1 V. This procedure was repeated until the voltage reached 1 V. The colors on the map correspond to the number of solitons, with oblique hatching indicating multipulsing regimes and solid filled areas corresponding to the HML regimes. Despite increasing the current and voltage, the maximum order of the achieved HML regimes did not exceed 9.

Next, we initiated the training procedure of the SAC algorithm. Each episode consisted with maximum 30 steps of adjusting the harmonic mode-locked regime. Different episodes took a different amount of time, depending on whether the mode-locked regime was maintained or not. The evolution of the learning curve of the SAC algorithm corresponding to the average reward obtained by the agent is depicted in Figure 6c. The training procedure lasted 45 h at our experiment. The process of tuning hyperparameters of the SAC algorithm, such as the number of layers of neural networks, experience buffer size, and other parameters, was a time-consuming task for our experimental setup. A possible solution would be to create a numerical model of the laser system in order to parallelize the training of different RL algorithms with different architectures of neural networks.

We investigated the behavior of the SAC algorithm during the first train procedure (blue line at Figure 6c). For the first 200 episodes, the agent’s actions exhibited chaotic behavior with a tendency to decrease the pump current, resulting in the loss of a mode-locked regime at the end of each episode. Consequently, the total reward gained by the agent during this period was nearly zero. However, starting from the 200th episode, the agent began to succeed in achieving a low-order HML regime, indicating a positive direction in adjusting the laser cavity and earning a high reward in specific episodes. Despite this progress, frequent transitions into Q-switched regimes kept the average reward low until the 500th episode.

Analyzing the significant increase in the average reward starting from the 500th episode, we identified that the value of the first pump current step is crucial for preserving the mode-locked regime. The initial large step in the pumping current tends to transition mode-locked regimes into a Q-switched regime, characterized by microsecond pulses with a repetition rate at the kHz scale. Once the Q-switched regime is initiated, reverting to the mode-locked regime becomes impossible without adjusting the pumping current to a prethreshold value. During the training procedure, a substantial amount of data allowed us to conduct a statistical analysis. In Figure 6b, the probability of the SAC algorithm making a certain step of the current is shown in red. The algorithm exhibits a higher probability of taking the first step with a value of 0.23 mA. To understand this choice, we also plotted the probabilities of transitioning to a Q-switched regime after a certain value of the first step (blue bars). This clearly illustrates that the algorithm learns to make the first step with the minimum probability of entering a Q-switched regime.

The drastic decrease in the reward curve around the 900th episode can be attributed to mechanical disturbances in the laser cavity during the training procedure. Nevertheless, the SAC algorithm demonstrated adaptability to the new conditions and successfully recovered the total reward. However, additional local decreases in the total reward can be observed. We implemented the SAC algorithm with a fixed parameter alpha 5, which compels the agent to explore new trajectories at the same rate. Exploration at a constant rate may lead to failure in the learning process. In our case, episodes end when a mode-locked regime is lost, resulting in zero rewards for an early transition to a Q-switched regime. Such failures continue to occur around the 1100th and 1300th episodes, but with a lesser decrease in the total reward. To control the agent’s research process, a modification of the SAC algorithm with an adapting temperature coefficient can be used [61].

The SAC algorithm have learned to get the HML regime with the 11th order at saturated part of the learning curve. The fluctuations in the average reward at the saturation part of the curve are caused by different sets of intermediate states before the HML regime with the highest order was obtained. Then, we tested the SAC algorithm by another two independent training procedures. All training procedures led to the possibility of the SAC algorithm to generate HML regimes with the highest order. After each training procedure, the SAC algorithm was tested in operation regime, which does not involve updating the weights of neural networks of the algorithm. The experiment shows that SAC algorithm achieves 11th order HML regime 10 out of 10 attempts. In the operation regime, the SAC algorithm took six steps on average to adjust voltage and current for the highest order, which took about a minute. A significant portion of time is required to wait until the pulsed regime has stabilized.

Dashed lines in Figure 6a represent different trajectories of the agent for various initializations. Although the trajectories do not coincide, they share a common pattern: at the beginning of the adjustment, the agent increases the voltage applied to the saturable absorber along with an increase in pump current. Toward the end, after achieving a relatively high order of the HML regime, the agent decreases the voltage on the SWCNTs. In contrast to the naive strategy of sequentially increasing both the voltage and the current, the SAC algorithm has identified a strategy for generating an HML regime with the highest order. At the final point, approximately 350 mA of current and 0.5 V yield an almost two-fold increase in the repetition rate of the pulse train compared to the mapping results. A video showing the oscilloscope trace of the output radiation during the adjustment of the HML regime by the SAC algorithm is available in the Supplementary Materials.

## 5 Conclusions

In conclusion, we demonstrate the performance of the SAC algorithm in learning the strategy to generate a harmonic mode-locked regime inside a mode-locked fiber laser. The algorithm was challenged to deal with a complex multistable system with various stable attractors, including Q-switched, single, and multisoliton regimes. We designed the architecture of the SAC algorithm, the state describing the laser system, and a reward function to efficiently train the algorithm and optimize its performance, relying only on oscilloscope data. Our findings indicate that the SAC algorithm possesses the capability to identify nontrivial sequences of steps for adjusting the cavity elements’ parameters, thereby guaranteeing the generation of the HML regime and maximizing its order.

The SAC algorithm includes training and operation stages, unlike evolutionary algorithms, which only have a single optimization stage. The training stage is time and data consuming, which may not be suitable or surplus for certain applications. But at the operation stage, the agent adjusts the desired regime fast based on the learned behavior of a system. With a time-varying system, like a multistable laser, the evolutionary algorithm must be optimized every restart of the system.

The present state of the field of mode-locked lasers is advancing toward multimode platforms, posing a challenge to the design and adjusting process [62]–[64]. There is no universal analytic explanation for many of these phenomena, and investigating their behavior requires enormous computational efforts. We anticipate that RL algorithms are a powerful tool for controlling nonlinear optical devices with strong multistability phenomena or when multiple physical effects interplay with each other.
